# Crystal structure and Hirshfeld surface analysis of (1*H*-imidazole-κ*N* ^3^)[4-methyl-2-({[2-oxido-5-(2-phenyl­diazen-1-yl)phen­yl]methyl­idene}amino)penta­noate-κ^3^ *O*,*N*,*O*′]copper(II)

**DOI:** 10.1107/S2056989024002986

**Published:** 2024-04-11

**Authors:** Ai Kaneda, Soma Suzuki, Daisuke Nakane, Yukiyasu Kashiwagi, Takashiro Akitsu

**Affiliations:** a Department of Chemistry, Faculty of Science, Tokyo University of Science, 1-3 Kagurazaka, Shinjuku-ku, Tokyo 162-8601, Japan; b Osaka Research Institute of Industrial Science and Technology, 1-6-50 Morinomiya, Joto-ku, Osaka 536-8553, Japan; Universidade Federal do ABC, Brazil

**Keywords:** Schiff base ligand, copper(II) complex, amino acid, Azo­benzene, Hirshfeld analysis, crystal structure

## Abstract

The title copper(II) complex, [Cu(C_18_H_19_N_3_O_3_)(C_3_H_4_N_2_)], consists of a tridentate ligand synthesized from l-leucine and azo­benzene-salicyl­aldehyde. One imidazole mol­ecule is additionally coordinated to the copper(II) ion in the equatorial plane. The crystal structure features N—H⋯O hydrogen bonds. A Hirshfeld surface analysis indicates that the most important contributions to the packing are from H⋯H (52.0%) and C⋯H/H⋯C (17.9%) contacts.

## Abstract 2

The crystal structure is reported of the copper(II) complex with an amino acid Schiff base ligand synthesized from azo­benzene-salicyl­aldehyde, l-leucine and copper(II) acetate. The crystal structure features N—H⋯O hydrogen bonds.

## 1. Chemical context

Azo compounds have been thoroughly explored due to their numerous uses in organic synthesis and high-tech fields such as liquid crystalline displays, lasers, leather, inkjet printers, dyeing textile fibers, optical data storage, optical switching technologies, and photo-refractive polymer industries (Andreini *et al.*, 2008 ▸; Stappen *et al.*, 2022 ▸). Furthermore, azo compounds have shown a wide range of pharmacological and medicinal potentials and can be employed as anti­bacterial, anti­fungal, anti­tumor, and anti­oxidant agents (Andreini *et al.*, 2008 ▸; Van Stappen *et al.*, 2022 ▸). Active azo group-containing ligands have been shown to possess a strong coordination ability with various metal ions in different oxidation states and form compounds with improved pharmacological characteristics (Dabis & Ward, 2019 ▸); these compounds are used in a variety of biological processes, such as the suppression of RNA and DNA as well as several anti­microbial activities (Stappen *et al.*, 2022 ▸; Dabis *et al.*, 2019 ▸). Over the past few decades, it has become clear that azo Schiff base compounds have a broad range of uses, particularly in the fields of biological applications and chemical synthesis, as well as in a number of industrial applications (Andreini *et al.*, 2008 ▸; Stappen *et al.*, 2022 ▸; Dabis & Ward, 2019 ▸). Furthermore, azo Schiff base compounds can form stable complexes with various metal ions, and find several applications in the treatment of nuclear waste, corrosion control, metal recovery, medicine, *etc* (Andreini *et al.*, 2008 ▸; Van Stappen *et al.*, 2022 ▸; Gandin *et al.*, 2013 ▸; Nishihara *et al.*, 2005 ▸).

On the other hand, copper has various oxidation states, of which the divalent oxidation state is the most stable. Copper(II) ions readily form complexes and produce abundant coordination chemistry, while amino acid Schiff base–copper(II) complexes have been studied in terms of photoreaction with titanium dioxide (Takeshita *et al.*, 2015 ▸), photocatalytic reduction of hexa­valent chromium (Nakagame *et al.*, 2019 ▸), and anti­bacterial activity (Otani *et al.*, 2022 ▸). The introduction of a hydroxyl group is effective in increasing solubility in aqueous solvents (Miyagawa *et al.*, 2020 ▸). In addition, similar complexes have been reported (Nejati *et al.*, 2019 ▸; Eren *et al.*, 2015 ▸; Zhang *et al.*, 2009 ▸). In this report, we describe the crystal structure and inter­molecular inter­actions of a leucine derivative copper(II) complex with an imidazole group. 
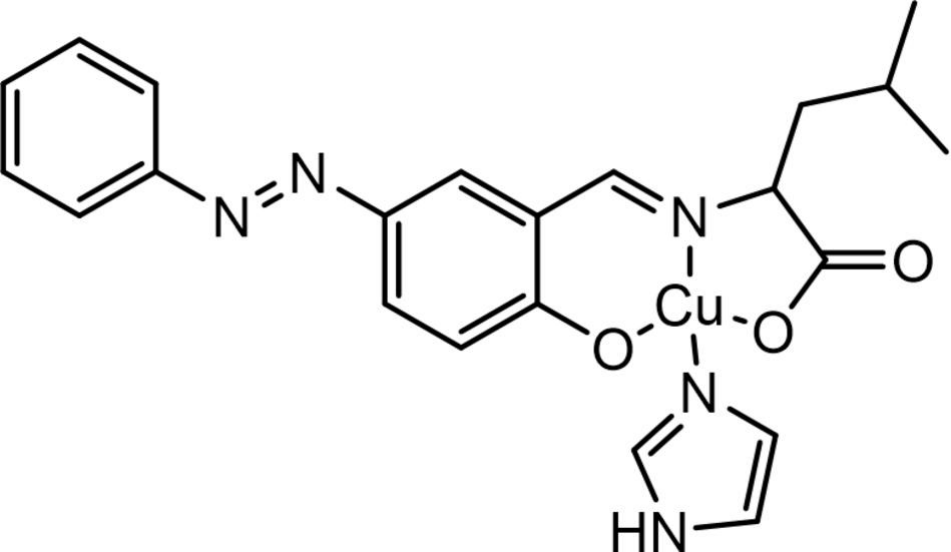


## 2. Structural commentary

**Figure 1:**
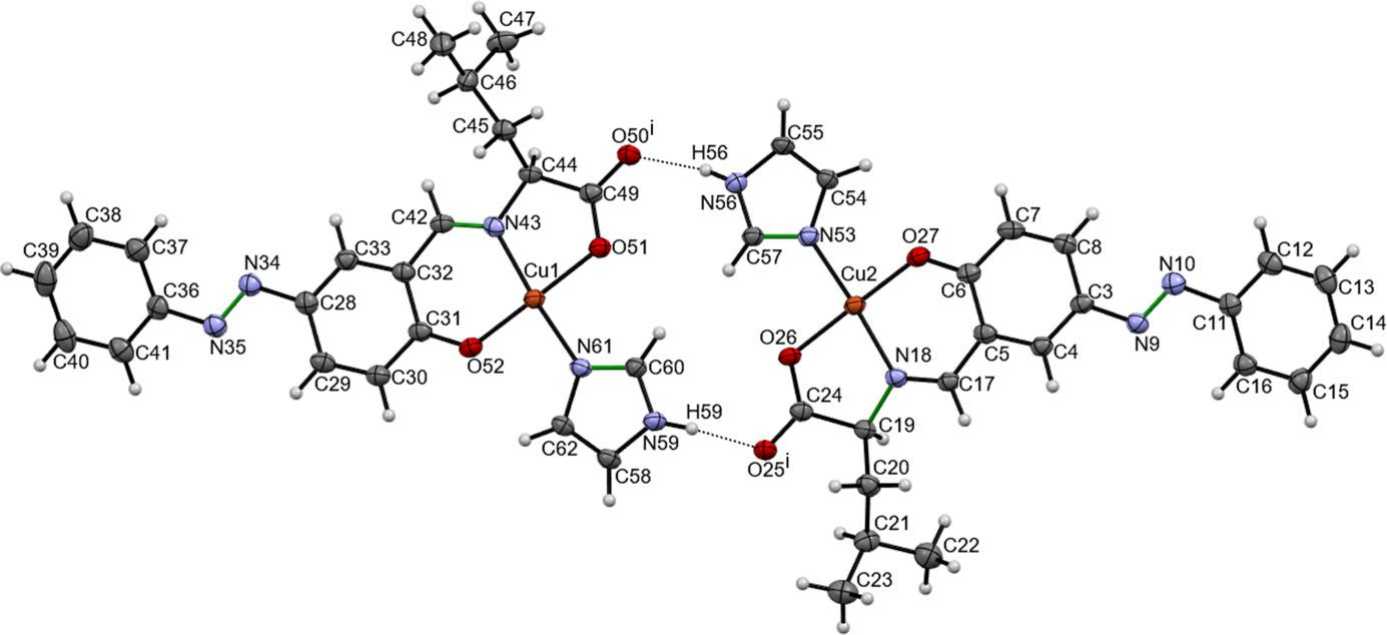
Mol­ecular structure of title compound. Displacement ellipsoids (non-H) are drawn at the 50% probability level, with H atoms presented as spheres. Dashed lines indicate inter­molecular hydrogen bonds. Double C=N or N=N bonds are drawn in green. [Symmetry code: (i) *x*, *y*, *z*]

The mol­ecular structure of the title compound consists of a tridentate ligand synthesized from l-leucine, azo­benzene-salicyl­aldehyde and one imidazole mol­ecule coordinating by the copper(II) ion (Fig. 1 ▸). The planarity of the π-electron system allows for the acquisition of large resonance energies due to the overlap of orbitals, resulting in a planar structure. The azo group is in a *trans* conformation.

Two independent mol­ecules are contained in an asymmetric unit, mol­ecule 1 (containing atom Cu1) and mol­ecule 2 (including Cu2). In mol­ecule 1, the C60=N61 double-bond distance is 1.315 (5) Å, close to a typical C=N double-bond length for an imine compounds. The Cu1—O51 and Cu1—O52 bonds lengths are 1.948 (3) and 1.896 (3) Å, respectively, close to a typical Cu—O bond length. The Cu1—N43 and Cu1—N61 bonds lengths of 1.936 (4) and 1.948 (4) Å corresponds to the typical Cu—N bond length (Katsuumi *et al.*, 2020 ▸).

Similarly, in mol­ecule 2 the C57=N53 double-bond distance is 1.334 (5) Å, close to a typical C=N double-bond length for an imine compounds (Katsuumi *et al.*, 2020 ▸). The Cu2—O26 and Cu2—O27 bonds lengths are 1.947 (3) and 1.904 (3) Å, respectively, close to a typical Cu—O bond length. The Cu2—N18 and Cu2—N53 bonds lengths of 1.928 (4) and 1.949 (4) Å corresponds to the typical Cu—N bond length.

## 3. Supra­molecular features

**Figure 2:**
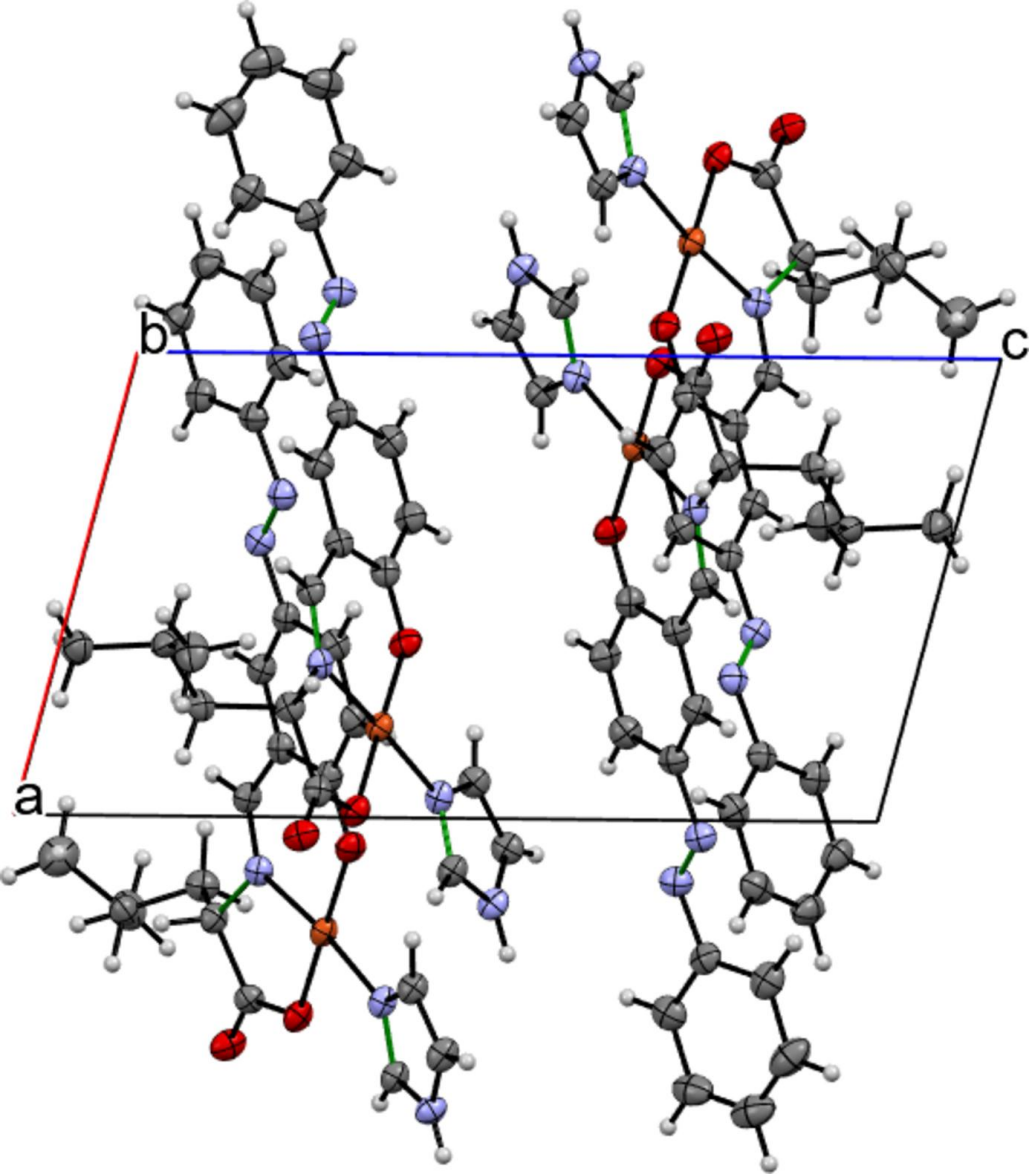
Crystal packing viewed down the crystallographic *b* axis. Double C=N or N=N bonds are drawn in green.

There are only two inter­molecular hydrogen bonds (O25⋯H59—N59 and O50⋯H56—N56) between the two mol­ecules in the asymmetric unit (Fig. 1 ▸ and Table 1 ▸). No other inter­molecular hydrogen bonds are found in the crystal packing (Fig. 2 ▸).

**Figure 3:**
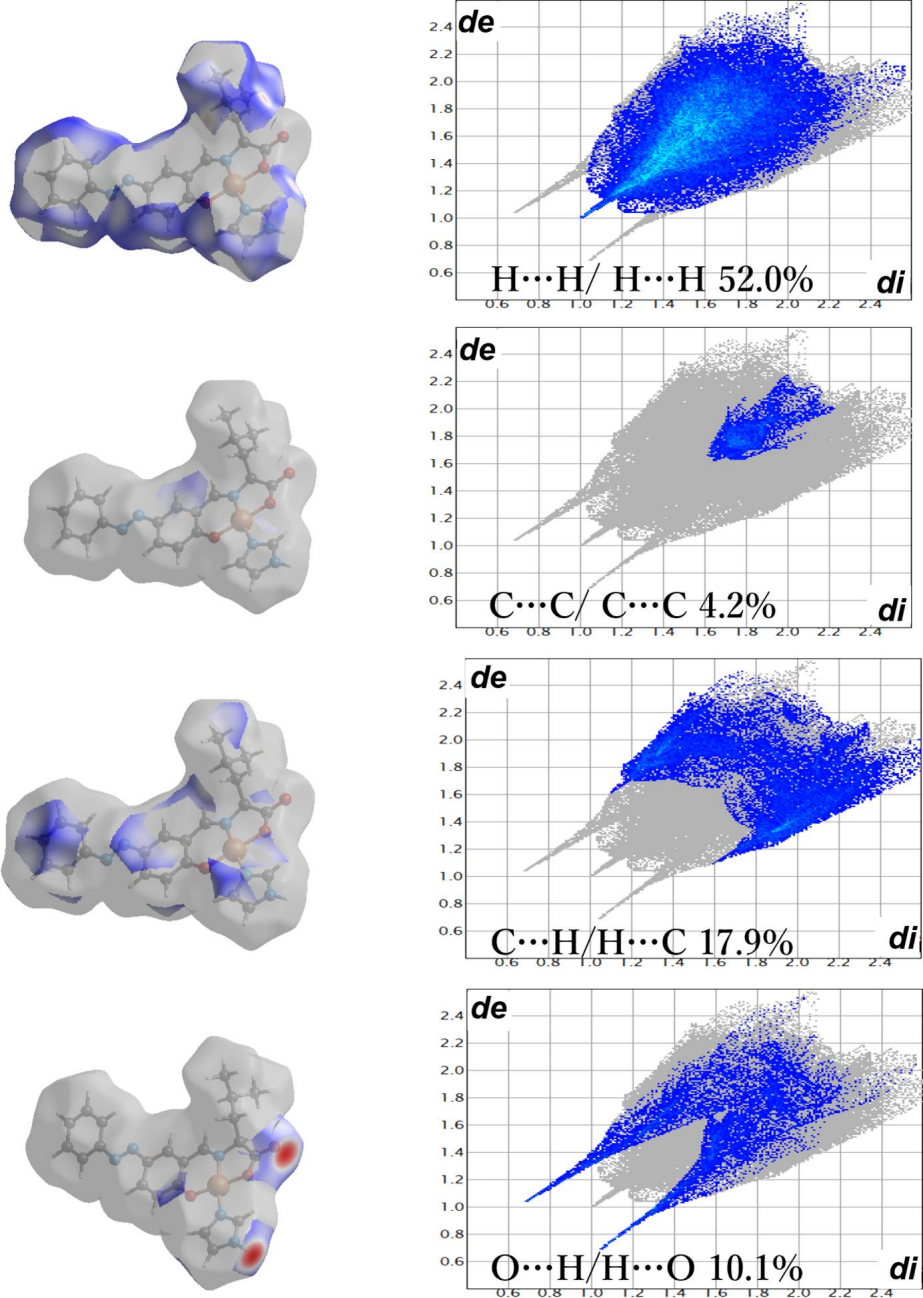
Hirshfeld surfaces mapped over *d*_norm_ and two-dimensional fingerprint plots.

A Hirshfeld surface analysis (Spackman & Jayatilaka, 2009 ▸; McKinnon *et al.*, 2007 ▸) was performed to further investigate the inter­molecular inter­actions and contacts. The inter­molecular O⋯H—O hydrogen bonds are indicated by bright-red spots appearing near O25 and O50 on the Hirshfeld surfaces mapped over *d*_norm_ and by two sharp spikes of almost the same length in the region 1.6 Å < (*d*_e_ + *d*_i_) < 2.0 Å in the 2D finger plots (Fig. 3 ▸). The contributions to the packing from H⋯H, C⋯C, C⋯H/H⋯C and H⋯O/O⋯H contacts are 52.0, 4.2, 17.9, and 10.1%, respectively. This structure is characterized by high proportions of H⋯H and C⋯H/H⋯C inter­actions, where H⋯H are van der Waals inter­actions. The high value of C⋯H/H⋯C is thought to arise from C—H⋯π inter­actions due to the presence of aromatic rings in the compound. The low value of C⋯C/C⋯C is the result of the low contribution of π–π stacking due to non-overlapping aromatic rings in the structure.

## 4. Database survey

A search in the Cambridge Structural Database (CSD, Version 5.41, update of January 2024; Groom *et al.*, 2016 ▸) for similar structures returned three relevant entries: [Cu(*L*1)_2_] and [Cu(*L*2)_2_] {H*L*1 = 4-[(*E*)-phenyl­diazen­yl]-2-[(*E*)-(propyl­imino)­meth­yl]phenol and H*L*2 = salicyl­idene­propyl­amine, the second structure was only calculated in the gas phase} (KODPOL; Nejati *et al.*, 2019 ▸), 4-[(*E*)-phenyl­diazen­yl]-2-[(*E*)-{[4-(propan-2-yl)phen­yl]imino}­meth­yl]phenol (H*L*) and its copper(II) complex (ZUHFUF; Eren *et al.*, 2015 ▸), (2,2′-bi­pyridine-κ^2^ *N*,*N*′){*N*-[2-oxido-5-(phenyl­diazen­yl)benzyl­idene-κ*O*]gly­cinato-κ^2^ *N*,*O*}copper(II) (QUCFIE; Zhang *et al.*, 2009 ▸).

## 5. Synthesis and crystallization

Azo­benzene-salicyl­aldehyde (226 mg, 1.00 mmol) and l-leu­cine (131 mg, 1.00 mmol) were dissolved in methanol (100 mL) and stirred at 313 K for 3 h to give a red solution. Copper(II) acetate monohydrate (199 mg, 1.00 mmol) was added and stirred for 1 h, and imidazole (68 mg, 1.00 mmol) was added and stirred for 2 h to give a dark-green solution. The reaction solution was allowed to stand at 298 K for 4 d to give a green powder, yield: 0.3507 g (74.9%). Recrystallization was performed by vapor diffusion of diethyl ether into a DMF solution of the copper(II) complex.

Elementary analysis: found: C, 56.23; H, 5.01; N, 14.79%. Calculated C_22_H_23_CuN_5_O_3_, C, 56.34; H, 4.94; N, 14.93%.

IR (KBr, cm^−1^): 3450 *br*, 2921 *m*, 2748 *br*, 1631 *s* (C=N double bond), 1607 *s* (C=O double bond), 1529 *w*, 1468 *m*, 1420 *m*, 1381 *s*, 1324 *w*, 1191 *w*, 1156 *w*, 1112 *m*, 1106 *m*, 835 *m*, 764 *m*, 691 *w*, 653 *w*, 529 *w* (Fig. S1 in the supporting information).

UV–vis: 261 nm (ɛ = 19000 M^−1^ cm^−1^, π–π^*^); 391 nm (ɛ = 22000 *M* ^−1^ cm^−1^, *n*–π^*^); 676 nm (ɛ = 135 *M* ^−1^ cm^−1^, *d*–*d*) (Figs. S2, S3).

CD: 253 nm (6.72 dm^3^ *M* ^−1^ cm^−1^), 334 nm (−0.57 dm^3^ *M* ^−1^ cm^−1^), 383 nm (−4.04 dm^3^ *M* ^−1^ cm^−1^) (Fig. S4).

## 6. Refinement

Crystal data, data collection and structure refinement details are summarized in Table 2 ▸. All C-bound H atoms were placed in geometrically calculated positions (C—H = 0.93–0.98 Å) and were constrained using a riding model with *U*_iso_(H) = 1.2*U*_eq_(C) for *R*_2_CH and *R*_3_CH H atoms and 1.5*U*_eq_(C) for the methyl H atoms. The N-bound H atoms, H56 and H59, were located based on a difference-Fourier map. H56 was refined freely as an isotropic atom, and H59 atom was refined with a distance restraint of N—H = 0.86±0.02 Å. One outlier ( 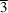 13 12) was omitted from the refinement.

## Supplementary Material

Crystal structure: contains datablock(s) I. DOI: 10.1107/S2056989024002986/ee2005sup1.cif

Structure factors: contains datablock(s) I. DOI: 10.1107/S2056989024002986/ee2005Isup2.hkl

Supporting information file. DOI: 10.1107/S2056989024002986/ee2005sup3.tif

Supporting information file. DOI: 10.1107/S2056989024002986/ee2005sup4.tif

Supporting information file. DOI: 10.1107/S2056989024002986/ee2005sup5.tif

Supporting information file. DOI: 10.1107/S2056989024002986/ee2005sup6.tif

CCDC reference: 2347500

Additional supporting information:  crystallographic information; 3D view; checkCIF report

## Figures and Tables

**Table 1:** Hydrogen-bond geometry (Å, °)

*D*—H⋯*A*	*D*—H	H⋯*A*	*D*⋯*A*	*D*—H⋯*A*
N56—H56⋯O50	0.83 (5)	1.92 (6)	2.730 (5)	169 (5)
N59—H59⋯O25	0.90 (4)	1.83 (4)	2.726 (5)	175 (3)
C55—H55⋯O25^i^	0.95	2.38	3.316 (5)	168
C58—H58⋯O50^ii^	0.95	2.35	3.208 (6)	150

Symmetry codes: (i) 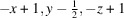 ; (ii) 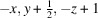 .

**Table 2:** Experimental details

Crystal data	
Chemical formula	[Cu(C_18_H_19_N_3_O_3_)(C_3_H_4_N_2_)]
*M*_r_	468.99
Crystal system, space group	Monoclinic, *P*2_1_
Temperature (K)	100
*a*, *b*, *c* (Å)	8.2816 (2), 17.4856 (3), 14.9186 (3)
β (°)	104.478 (2)
*V* (Å^3^)	2091.74 (8)
*Z*	4
Radiation type	Cu *K*α
μ (mm^−1^)	1.77
Crystal size (mm)	0.35 × 0.05 × 0.03

Data collection	
Diffractometer	XtaLAB Synergy, Dualflex, HyPix
Absorption correction	Multi-scan (*CrysAlis PRO*; Rigaku OD, 2023 ▸)
*T*_min_, *T*_max_	0.338, 1.000
No. of measured, independent and observed [*I* > 2σ(*I*)] reflections	34288, 7772, 7186
*R*_int_	0.060
(sin θ/λ)_max_ (Å^−1^)	0.632

Refinement	
*R*[*F* ^2^ > 2σ(*F* ^2^)], *wR*(*F* ^2^), *S*	0.036, 0.096, 1.05
No. of reflections	7772
No. of parameters	571
No. of restraints	2
H-atom treatment	H atoms treated by a mixture of independent and constrained refinement
Δρ_max_, Δρ_min_ (e Å^−3^)	0.35, −0.38
Absolute structure	Flack *x* determined using 3052 quotients [(*I* ^+^)−(*I* ^−^)]/[(*I* ^+^)+(*I* ^−^)] (Parsons *et al.*, 2013 ▸)
Absolute structure parameter	0.012 (17)

Computer programs: *CrysAlis PRO* (Rigaku OD, 2023 ▸), *SHELXT2018/2* (Sheldrick, 2015*a* ▸), *SHELXL2018/3* (Sheldrick, 2015*b* ▸) and *OLEX2* (Dolomanov *et al.*, 2009 ▸).

## supplementary crystallographic information

### Crystal data

**Table:**

[Cu(C_18_H_19_N_3_O_3_)(C_3_H_4_N_2_)]	*F*(000) = 972
*M**_r_* = 468.99	*D*_x_ = 1.489 Mg m^−^^3^
Monoclinic, *P*2_1_	Cu *K*α radiation, λ = 1.54184 Å
*a* = 8.2816 (2) Å	Cell parameters from 21230 reflections
*b* = 17.4856 (3) Å	θ = 3.9–77.0°
*c* = 14.9186 (3) Å	µ = 1.77 mm^−^^1^
β = 104.478 (2)°	*T* = 100 K
*V* = 2091.74 (8) Å^3^	Block, green
*Z* = 4	0.35 × 0.05 × 0.03 mm

### Data collection

**Table:**

XtaLAB Synergy, Dualflex, HyPix diffractometer	7772 independent reflections
Radiation source: micro-focus sealed X-ray tube, PhotonJet (Cu) X-ray Source	7186 reflections with *I* > 2σ(*I*)
Mirror monochromator	*R*_int_ = 0.060
Detector resolution: 10.0000 pixels mm^-1^	θ_max_ = 77.1°, θ_min_ = 3.1°
ω scans	*h* = −9→10
Absorption correction: multi-scan (CrysAlisPro; Rigaku OD, 2023)	*k* = −21→18
*T*_min_ = 0.338, *T*_max_ = 1.000	*l* = −18→17
34288 measured reflections	

### Refinement

**Table:**

Refinement on *F*^2^	Hydrogen site location: mixed
Least-squares matrix: full	H atoms treated by a mixture of independent and constrained refinement
*R*[*F*^2^ > 2σ(*F*^2^)] = 0.036	*w* = 1/[σ^2^(*F*_o_^2^) + (0.0594*P*)^2^ + 0.213*P*] where *P* = (*F*_o_^2^ + 2*F*_c_^2^)/3
*wR*(*F*^2^) = 0.096	(Δ/σ)_max_ < 0.001
*S* = 1.05	Δρ_max_ = 0.35 e Å^−^^3^
7772 reflections	Δρ_min_ = −0.38 e Å^−^^3^
571 parameters	Absolute structure: Flack *x* determined using 3052 quotients [(*I*^+^)-(*I*^-^)]/[(*I*^+^)+(*I*^-^)] (Parsons *et al.*, 2013)
2 restraints	Absolute structure parameter: 0.012 (17)
Primary atom site location: dual	

### Special details

**Table:**

Geometry. All esds (except the esd in the dihedral angle between two l.s. planes) are estimated using the full covariance matrix. The cell esds are taken into account individually in the estimation of esds in distances, angles and torsion angles; correlations between esds in cell parameters are only used when they are defined by crystal symmetry. An approximate (isotropic) treatment of cell esds is used for estimating esds involving l.s. planes.
Refinement. 1. Fixed Uiso At 1.2 times of: All C(H) groups, All C(H,H) groups At 1.5 times of: All C(H,H,H) groups 2. Restrained distances H59-N59 0.86 with sigma of 0.02 3.a Ternary CH refined with riding coordinates: C44(H44), C46(H46), C19(H19), C21(H21) 3.b Secondary CH2 refined with riding coordinates: C45(H45A,H45B), C20(H20A,H20B) 3.c Aromatic/amide H refined with riding coordinates: C29(H29), C30(H30), C33(H33), C37(H37), C38(H38), C39(H39), C40(H40), C41(H41), C42(H42), C58(H58), C60(H60), C62(H62), C4(H4), C7(H7), C8(H8), C12(H12), C13(H13), C14(H14), C15(H15), C16(H16), C17(H17), C54(H54), C55(H55), C57(H57) 3.d Idealised Me refined as rotating group: C47(H47A,H47B,H47C), C48(H48A,H48B,H48C), C22(H22A,H22B,H22C), C23(H23A,H23B, H23C)

### Fractional atomic coordinates and isotropic or equivalent isotropic displacement parameters (Å^2^)

**Table:**

	*x*	*y*	*z*	*U*_iso_*/*U*_eq_	
Cu1	−0.19509 (7)	0.50887 (3)	0.39555 (4)	0.02421 (15)	
C28	−0.8701 (5)	0.5618 (3)	0.2550 (3)	0.0270 (9)	
C29	−0.8112 (6)	0.6217 (3)	0.3190 (3)	0.0301 (9)	
H29	−0.885387	0.660360	0.328895	0.036*	
C30	−0.6467 (5)	0.6233 (3)	0.3665 (3)	0.0302 (9)	
H30	−0.608958	0.663525	0.409437	0.036*	
C31	−0.5302 (5)	0.5670 (3)	0.3540 (3)	0.0256 (8)	
C32	−0.5918 (5)	0.5059 (3)	0.2914 (3)	0.0248 (8)	
C33	−0.7612 (5)	0.5059 (3)	0.2436 (3)	0.0265 (8)	
H33	−0.801844	0.465329	0.201644	0.032*	
N34	−1.0357 (4)	0.5564 (2)	0.2000 (2)	0.0280 (8)	
N35	−1.1322 (4)	0.6080 (2)	0.2144 (2)	0.0296 (8)	
C36	−1.2973 (5)	0.6037 (3)	0.1572 (3)	0.0289 (9)	
C37	−1.3437 (6)	0.5574 (3)	0.0783 (3)	0.0356 (10)	
H37	−1.263192	0.526977	0.059262	0.043*	
C38	−1.5093 (7)	0.5566 (3)	0.0285 (4)	0.0456 (12)	
H38	−1.542498	0.524603	−0.024422	0.055*	
C39	−1.6264 (6)	0.6015 (3)	0.0545 (4)	0.0459 (13)	
H39	−1.739347	0.599988	0.019703	0.055*	
C40	−1.5807 (6)	0.6489 (3)	0.1309 (4)	0.0407 (12)	
H40	−1.661227	0.680543	0.148084	0.049*	
C41	−1.4158 (6)	0.6497 (3)	0.1821 (3)	0.0336 (10)	
H41	−1.383673	0.681968	0.234717	0.040*	
C42	−0.4887 (5)	0.4450 (3)	0.2737 (3)	0.0256 (8)	
H42	−0.542002	0.405534	0.233279	0.031*	
N43	−0.3304 (4)	0.4389 (2)	0.3073 (2)	0.0235 (7)	
C44	−0.2358 (5)	0.3729 (2)	0.2858 (3)	0.0253 (8)	
H44	−0.285929	0.325257	0.304277	0.030*	
C45	−0.2385 (5)	0.3665 (2)	0.1821 (3)	0.0268 (8)	
H45A	−0.253417	0.418480	0.155125	0.032*	
H45B	−0.127757	0.347960	0.177695	0.032*	
C46	−0.3704 (5)	0.3149 (3)	0.1227 (3)	0.0299 (9)	
H46	−0.482471	0.333867	0.126253	0.036*	
C47	−0.3528 (6)	0.2325 (3)	0.1559 (3)	0.0389 (11)	
H47A	−0.238563	0.214892	0.160594	0.058*	
H47B	−0.431190	0.200207	0.111696	0.058*	
H47C	−0.377136	0.229136	0.216733	0.058*	
C48	−0.3602 (6)	0.3200 (3)	0.0221 (3)	0.0375 (10)	
H48A	−0.380558	0.372884	0.000368	0.056*	
H48B	−0.444536	0.286450	−0.016358	0.056*	
H48C	−0.249088	0.304030	0.017603	0.056*	
C49	−0.0567 (5)	0.3787 (3)	0.3436 (3)	0.0255 (8)	
O50	0.0400 (4)	0.32582 (18)	0.3384 (2)	0.0306 (7)	
O51	−0.0141 (3)	0.43815 (18)	0.39377 (18)	0.0275 (6)	
O52	−0.3762 (4)	0.57403 (18)	0.3994 (2)	0.0296 (6)	
C58	0.0637 (5)	0.6684 (3)	0.5806 (3)	0.0275 (9)	
H58	0.077112	0.714775	0.614874	0.033*	
N59	0.1840 (5)	0.6146 (2)	0.5812 (2)	0.0269 (7)	
H59	0.292 (4)	0.613 (3)	0.613 (3)	0.033 (13)*	
C60	0.1134 (5)	0.5586 (3)	0.5240 (3)	0.0256 (9)	
H60	0.170091	0.513940	0.512245	0.031*	
N61	−0.0447 (4)	0.5731 (2)	0.4861 (2)	0.0254 (7)	
C62	−0.0784 (5)	0.6427 (2)	0.5217 (3)	0.0268 (9)	
H62	−0.183019	0.668219	0.507303	0.032*	
Cu2	0.75324 (7)	0.44616 (3)	0.60625 (4)	0.02372 (15)	
C3	1.4299 (5)	0.3972 (2)	0.7467 (3)	0.0254 (8)	
C4	1.3185 (5)	0.4517 (3)	0.7593 (3)	0.0246 (8)	
H4	1.357625	0.492203	0.801558	0.029*	
C5	1.1485 (5)	0.4500 (3)	0.7123 (2)	0.0248 (8)	
C6	1.0894 (5)	0.3893 (3)	0.6476 (3)	0.0251 (8)	
C7	1.2091 (6)	0.3346 (3)	0.6350 (3)	0.0309 (10)	
H7	1.173286	0.294233	0.591985	0.037*	
C8	1.3723 (5)	0.3377 (3)	0.6818 (3)	0.0272 (9)	
H8	1.447968	0.299988	0.671126	0.033*	
N9	1.5951 (4)	0.4054 (2)	0.8008 (2)	0.0263 (7)	
N10	1.6950 (5)	0.3548 (2)	0.7891 (2)	0.0288 (8)	
C11	1.8613 (5)	0.3639 (3)	0.8464 (3)	0.0270 (9)	
C12	1.9794 (6)	0.3131 (3)	0.8308 (3)	0.0316 (9)	
H12	1.947320	0.274106	0.785450	0.038*	
C13	2.1446 (6)	0.3187 (3)	0.8810 (3)	0.0355 (10)	
H13	2.225310	0.283677	0.870132	0.043*	
C14	2.1912 (6)	0.3757 (3)	0.9470 (3)	0.0363 (10)	
H14	2.304408	0.380317	0.980622	0.044*	
C15	2.0729 (6)	0.4261 (3)	0.9641 (3)	0.0337 (10)	
H15	2.105191	0.464577	1.010017	0.040*	
C16	1.9072 (6)	0.4205 (3)	0.9143 (3)	0.0298 (9)	
H16	1.826070	0.454794	0.926238	0.036*	
C17	1.0411 (5)	0.5061 (3)	0.7364 (3)	0.0245 (8)	
H17	1.090490	0.541958	0.783152	0.029*	
N18	0.8835 (4)	0.5123 (2)	0.7004 (2)	0.0234 (7)	
C19	0.7810 (5)	0.5666 (3)	0.7377 (3)	0.0261 (8)	
H19	0.767695	0.543882	0.796933	0.031*	
C20	0.8504 (5)	0.6468 (3)	0.7612 (3)	0.0302 (9)	
H20A	0.811339	0.680367	0.706561	0.036*	
H20B	0.973736	0.644989	0.775757	0.036*	
C21	0.7961 (5)	0.6811 (3)	0.8440 (3)	0.0310 (9)	
H21	0.680032	0.663487	0.841133	0.037*	
C22	0.9113 (7)	0.6542 (3)	0.9365 (4)	0.0460 (12)	
H22A	0.917061	0.598207	0.937339	0.069*	
H22B	0.866785	0.671948	0.987867	0.069*	
H22C	1.023295	0.675310	0.943143	0.069*	
C23	0.7960 (7)	0.7675 (3)	0.8392 (3)	0.0414 (11)	
H23A	0.907688	0.785571	0.838557	0.062*	
H23B	0.764470	0.788535	0.893309	0.062*	
H23C	0.715705	0.784349	0.782637	0.062*	
C24	0.6064 (5)	0.5679 (2)	0.6714 (3)	0.0250 (8)	
O25	0.5075 (4)	0.61784 (18)	0.6821 (2)	0.0320 (7)	
O26	0.5709 (3)	0.51539 (19)	0.61010 (18)	0.0275 (6)	
O27	0.9361 (4)	0.38167 (19)	0.60141 (19)	0.0292 (6)	
N53	0.6027 (4)	0.3813 (2)	0.5165 (2)	0.0253 (7)	
C54	0.6325 (5)	0.3094 (3)	0.4859 (3)	0.0265 (8)	
H54	0.736973	0.283759	0.501157	0.032*	
C55	0.4897 (5)	0.2811 (2)	0.4306 (3)	0.0281 (9)	
H55	0.475091	0.232852	0.400418	0.034*	
N56	0.3702 (5)	0.3362 (2)	0.4271 (2)	0.0271 (7)	
H56	0.273 (7)	0.327 (3)	0.399 (3)	0.029 (13)*	
C57	0.4412 (5)	0.3947 (2)	0.4791 (3)	0.0252 (9)	
H57	0.384538	0.440053	0.488501	0.030*	

### Atomic displacement parameters (Å^2^)

**Table:**

	*U*^11^	*U*^22^	*U*^33^	*U*^12^	*U*^13^	*U*^23^
Cu1	0.0283 (3)	0.0198 (3)	0.0230 (3)	−0.0007 (2)	0.0036 (2)	−0.0022 (2)
C28	0.028 (2)	0.024 (2)	0.030 (2)	−0.0025 (16)	0.0081 (16)	0.0033 (17)
C29	0.035 (2)	0.021 (2)	0.035 (2)	−0.0015 (17)	0.0105 (18)	−0.0019 (18)
C30	0.033 (2)	0.025 (2)	0.032 (2)	−0.0001 (17)	0.0075 (17)	−0.0051 (18)
C31	0.031 (2)	0.019 (2)	0.027 (2)	−0.0028 (16)	0.0081 (16)	−0.0007 (17)
C32	0.028 (2)	0.020 (2)	0.0268 (18)	−0.0020 (17)	0.0079 (14)	0.0003 (18)
C33	0.030 (2)	0.022 (2)	0.0280 (19)	−0.0023 (18)	0.0063 (15)	−0.0010 (19)
N34	0.0283 (19)	0.024 (2)	0.0307 (18)	−0.0008 (14)	0.0064 (14)	0.0017 (15)
N35	0.0285 (19)	0.025 (2)	0.0348 (19)	−0.0011 (14)	0.0077 (14)	0.0010 (15)
C36	0.032 (2)	0.023 (2)	0.030 (2)	−0.0003 (17)	0.0052 (17)	0.0080 (17)
C37	0.044 (3)	0.025 (3)	0.035 (2)	0.0008 (19)	0.0055 (19)	0.0044 (19)
C38	0.051 (3)	0.033 (3)	0.043 (3)	−0.003 (2)	−0.007 (2)	0.005 (2)
C39	0.036 (3)	0.040 (3)	0.056 (3)	−0.003 (2)	−0.001 (2)	0.021 (2)
C40	0.032 (2)	0.036 (3)	0.054 (3)	0.006 (2)	0.011 (2)	0.018 (2)
C41	0.037 (2)	0.025 (3)	0.041 (2)	0.0006 (18)	0.0128 (19)	0.0062 (19)
C42	0.030 (2)	0.020 (2)	0.0267 (19)	−0.0036 (18)	0.0076 (15)	−0.0041 (18)
N43	0.0304 (18)	0.0182 (18)	0.0227 (15)	0.0020 (14)	0.0084 (12)	0.0034 (14)
C44	0.030 (2)	0.018 (2)	0.0283 (19)	−0.0014 (15)	0.0078 (16)	−0.0037 (16)
C45	0.032 (2)	0.022 (2)	0.0273 (19)	−0.0021 (16)	0.0096 (16)	0.0014 (16)
C46	0.0242 (19)	0.035 (3)	0.028 (2)	0.0006 (17)	0.0024 (16)	−0.0039 (18)
C47	0.052 (3)	0.029 (3)	0.037 (2)	−0.013 (2)	0.013 (2)	−0.007 (2)
C48	0.038 (2)	0.044 (3)	0.030 (2)	0.006 (2)	0.0079 (18)	−0.005 (2)
C49	0.033 (2)	0.019 (2)	0.0254 (19)	−0.0032 (16)	0.0078 (16)	0.0009 (16)
O50	0.0301 (16)	0.0206 (17)	0.0385 (16)	0.0001 (12)	0.0038 (12)	−0.0048 (12)
O51	0.0324 (15)	0.0223 (16)	0.0256 (13)	−0.0002 (12)	0.0032 (11)	−0.0026 (12)
O52	0.0295 (16)	0.0252 (17)	0.0314 (15)	−0.0021 (12)	0.0028 (12)	−0.0071 (13)
C58	0.032 (2)	0.018 (2)	0.033 (2)	0.0000 (16)	0.0088 (17)	−0.0022 (17)
N59	0.032 (2)	0.0188 (19)	0.0282 (17)	−0.0022 (13)	0.0035 (14)	−0.0036 (14)
C60	0.032 (2)	0.019 (2)	0.025 (2)	0.0006 (16)	0.0031 (15)	0.0000 (16)
N61	0.034 (2)	0.0180 (19)	0.0237 (17)	0.0032 (14)	0.0055 (14)	−0.0014 (14)
C62	0.031 (2)	0.019 (2)	0.031 (2)	0.0020 (16)	0.0073 (16)	−0.0005 (17)
Cu2	0.0282 (3)	0.0192 (3)	0.0224 (3)	−0.0023 (2)	0.0037 (2)	−0.0022 (2)
C3	0.031 (2)	0.022 (2)	0.0226 (18)	−0.0042 (16)	0.0069 (15)	−0.0006 (16)
C4	0.030 (2)	0.019 (2)	0.0238 (17)	−0.0030 (16)	0.0048 (14)	−0.0002 (17)
C5	0.032 (2)	0.018 (2)	0.0250 (18)	−0.0022 (18)	0.0086 (15)	0.0018 (18)
C6	0.030 (2)	0.023 (2)	0.0230 (18)	−0.0038 (16)	0.0086 (16)	−0.0009 (16)
C7	0.037 (2)	0.024 (2)	0.033 (2)	−0.0052 (17)	0.0121 (18)	−0.0094 (19)
C8	0.031 (2)	0.022 (2)	0.030 (2)	0.0006 (16)	0.0097 (17)	−0.0011 (17)
N9	0.0297 (19)	0.021 (2)	0.0275 (17)	−0.0009 (14)	0.0059 (14)	0.0007 (14)
N10	0.0318 (19)	0.027 (2)	0.0283 (17)	−0.0014 (14)	0.0084 (14)	0.0008 (14)
C11	0.028 (2)	0.024 (2)	0.029 (2)	−0.0013 (16)	0.0084 (16)	0.0049 (16)
C12	0.039 (3)	0.024 (2)	0.034 (2)	0.0017 (18)	0.0130 (19)	0.0059 (18)
C13	0.036 (2)	0.032 (3)	0.040 (2)	0.0053 (19)	0.0131 (19)	0.013 (2)
C14	0.032 (2)	0.040 (3)	0.034 (2)	−0.0010 (19)	0.0029 (18)	0.011 (2)
C15	0.037 (2)	0.032 (3)	0.029 (2)	−0.0026 (17)	0.0025 (17)	0.0039 (17)
C16	0.037 (2)	0.024 (2)	0.029 (2)	0.0017 (16)	0.0081 (17)	0.0026 (16)
C17	0.030 (2)	0.019 (2)	0.0239 (17)	−0.0032 (17)	0.0058 (14)	−0.0015 (17)
N18	0.0284 (18)	0.0168 (17)	0.0251 (15)	−0.0022 (14)	0.0068 (12)	0.0008 (15)
C19	0.030 (2)	0.022 (2)	0.0255 (19)	0.0018 (16)	0.0057 (16)	−0.0034 (16)
C20	0.031 (2)	0.023 (2)	0.036 (2)	−0.0009 (16)	0.0084 (17)	−0.0004 (17)
C21	0.032 (2)	0.032 (3)	0.031 (2)	−0.0044 (18)	0.0100 (17)	−0.0040 (18)
C22	0.051 (3)	0.035 (3)	0.049 (3)	0.002 (2)	0.007 (2)	−0.002 (2)
C23	0.049 (3)	0.033 (3)	0.044 (3)	0.000 (2)	0.015 (2)	−0.006 (2)
C24	0.027 (2)	0.020 (2)	0.028 (2)	−0.0031 (16)	0.0071 (16)	−0.0013 (17)
O25	0.0311 (16)	0.0235 (17)	0.0374 (16)	0.0013 (12)	0.0012 (12)	−0.0070 (13)
O26	0.0329 (15)	0.0216 (16)	0.0252 (13)	−0.0017 (12)	0.0022 (11)	−0.0062 (12)
O27	0.0297 (16)	0.0278 (17)	0.0283 (14)	−0.0035 (12)	0.0041 (12)	−0.0081 (13)
N53	0.0316 (19)	0.0198 (19)	0.0232 (16)	−0.0015 (14)	0.0042 (13)	−0.0008 (14)
C54	0.031 (2)	0.019 (2)	0.027 (2)	0.0013 (16)	0.0044 (16)	−0.0008 (16)
C55	0.037 (2)	0.015 (2)	0.029 (2)	−0.0010 (16)	0.0027 (17)	−0.0024 (16)
N56	0.0264 (19)	0.024 (2)	0.0283 (18)	−0.0017 (14)	0.0019 (14)	−0.0024 (14)
C57	0.031 (2)	0.019 (2)	0.0237 (19)	−0.0007 (16)	0.0038 (16)	−0.0017 (16)

### Geometric parameters (Å, º)

**Table:**

Cu1—N43	1.936 (4)	Cu2—N18	1.928 (4)
Cu1—O51	1.948 (3)	Cu2—O26	1.948 (3)
Cu1—O52	1.896 (3)	Cu2—O27	1.904 (3)
Cu1—N61	1.948 (4)	Cu2—N53	1.949 (4)
C28—C29	1.418 (6)	C3—C4	1.371 (6)
C28—C33	1.370 (6)	C3—C8	1.421 (6)
C28—N34	1.415 (5)	C3—N9	1.411 (5)
C29—H29	0.9500	C4—H4	0.9500
C29—C30	1.370 (6)	C4—C5	1.408 (6)
C30—H30	0.9500	C5—C6	1.435 (6)
C30—C31	1.423 (6)	C5—C17	1.430 (6)
C31—C32	1.426 (6)	C6—C7	1.424 (6)
C31—O52	1.292 (5)	C6—O27	1.291 (5)
C32—C33	1.405 (6)	C7—H7	0.9500
C32—C42	1.430 (6)	C7—C8	1.359 (6)
C33—H33	0.9500	C8—H8	0.9500
N34—N35	1.259 (5)	N9—N10	1.253 (5)
N35—C36	1.421 (6)	N10—C11	1.436 (6)
C36—C37	1.401 (7)	C11—C12	1.383 (6)
C36—C41	1.390 (7)	C11—C16	1.400 (6)
C37—H37	0.9500	C12—H12	0.9500
C37—C38	1.387 (7)	C12—C13	1.390 (7)
C38—H38	0.9500	C13—H13	0.9500
C38—C39	1.377 (8)	C13—C14	1.386 (7)
C39—H39	0.9500	C14—H14	0.9500
C39—C40	1.383 (8)	C14—C15	1.388 (7)
C40—H40	0.9500	C15—H15	0.9500
C40—C41	1.388 (7)	C15—C16	1.391 (6)
C41—H41	0.9500	C16—H16	0.9500
C42—H42	0.9500	C17—H17	0.9500
C42—N43	1.285 (5)	C17—N18	1.286 (5)
N43—C44	1.474 (5)	N18—C19	1.471 (5)
C44—H44	1.0000	C19—H19	1.0000
C44—C45	1.545 (5)	C19—C20	1.524 (6)
C44—C49	1.522 (6)	C19—C24	1.535 (6)
C45—H45A	0.9900	C20—H20A	0.9900
C45—H45B	0.9900	C20—H20B	0.9900
C45—C46	1.520 (6)	C20—C21	1.538 (6)
C46—H46	1.0000	C21—H21	1.0000
C46—C47	1.518 (7)	C21—C22	1.543 (7)
C46—C48	1.527 (6)	C21—C23	1.512 (7)
C47—H47A	0.9800	C22—H22A	0.9800
C47—H47B	0.9800	C22—H22B	0.9800
C47—H47C	0.9800	C22—H22C	0.9800
C48—H48A	0.9800	C23—H23A	0.9800
C48—H48B	0.9800	C23—H23B	0.9800
C48—H48C	0.9800	C23—H23C	0.9800
C49—O50	1.239 (5)	C24—O25	1.234 (5)
C49—O51	1.277 (5)	C24—O26	1.277 (5)
C58—H58	0.9500	N53—C54	1.380 (6)
C58—N59	1.368 (6)	N53—C57	1.334 (5)
C58—C62	1.357 (6)	C54—H54	0.9500
N59—H59	0.90 (3)	C54—C55	1.355 (6)
N59—C60	1.334 (6)	C55—H55	0.9500
C60—H60	0.9500	C55—N56	1.372 (6)
C60—N61	1.315 (5)	N56—H56	0.82 (5)
N61—C62	1.385 (6)	N56—C57	1.328 (6)
C62—H62	0.9500	C57—H57	0.9500
			
N43—Cu1—O51	84.61 (13)	N18—Cu2—O26	84.40 (13)
N43—Cu1—N61	175.37 (15)	N18—Cu2—N53	174.23 (14)
O52—Cu1—N43	94.16 (13)	O26—Cu2—N53	90.83 (14)
O52—Cu1—O51	177.38 (14)	O27—Cu2—N18	94.44 (14)
O52—Cu1—N61	90.35 (13)	O27—Cu2—O26	177.84 (14)
N61—Cu1—O51	90.84 (13)	O27—Cu2—N53	90.43 (14)
C33—C28—C29	119.0 (4)	C4—C3—C8	118.6 (4)
C33—C28—N34	116.9 (4)	C4—C3—N9	116.2 (4)
N34—C28—C29	124.1 (4)	N9—C3—C8	125.2 (4)
C28—C29—H29	120.2	C3—C4—H4	118.6
C30—C29—C28	119.6 (4)	C3—C4—C5	122.8 (4)
C30—C29—H29	120.2	C5—C4—H4	118.6
C29—C30—H30	118.8	C4—C5—C6	118.7 (4)
C29—C30—C31	122.5 (4)	C4—C5—C17	117.9 (4)
C31—C30—H30	118.8	C17—C5—C6	123.2 (4)
C30—C31—C32	117.5 (4)	C7—C6—C5	117.0 (4)
O52—C31—C30	118.5 (4)	O27—C6—C5	124.1 (4)
O52—C31—C32	124.0 (4)	O27—C6—C7	118.9 (4)
C31—C32—C42	123.1 (4)	C6—C7—H7	118.6
C33—C32—C31	118.7 (4)	C8—C7—C6	122.8 (4)
C33—C32—C42	118.2 (4)	C8—C7—H7	118.6
C28—C33—C32	122.8 (4)	C3—C8—H8	120.0
C28—C33—H33	118.6	C7—C8—C3	120.1 (4)
C32—C33—H33	118.6	C7—C8—H8	120.0
N35—N34—C28	114.7 (4)	N10—N9—C3	115.4 (4)
N34—N35—C36	114.6 (4)	N9—N10—C11	114.1 (4)
C37—C36—N35	123.8 (4)	C12—C11—N10	116.2 (4)
C41—C36—N35	116.5 (4)	C12—C11—C16	120.0 (4)
C41—C36—C37	119.7 (4)	C16—C11—N10	123.7 (4)
C36—C37—H37	120.6	C11—C12—H12	119.8
C38—C37—C36	118.9 (5)	C11—C12—C13	120.4 (4)
C38—C37—H37	120.6	C13—C12—H12	119.8
C37—C38—H38	119.5	C12—C13—H13	120.2
C39—C38—C37	121.0 (5)	C14—C13—C12	119.7 (4)
C39—C38—H38	119.5	C14—C13—H13	120.2
C38—C39—H39	119.8	C13—C14—H14	119.9
C38—C39—C40	120.5 (5)	C13—C14—C15	120.2 (4)
C40—C39—H39	119.8	C15—C14—H14	119.9
C39—C40—H40	120.4	C14—C15—H15	119.8
C39—C40—C41	119.2 (5)	C14—C15—C16	120.3 (4)
C41—C40—H40	120.4	C16—C15—H15	119.8
C36—C41—H41	119.6	C11—C16—H16	120.3
C40—C41—C36	120.7 (5)	C15—C16—C11	119.3 (4)
C40—C41—H41	119.6	C15—C16—H16	120.3
C32—C42—H42	117.0	C5—C17—H17	117.2
N43—C42—C32	126.0 (4)	N18—C17—C5	125.6 (4)
N43—C42—H42	117.0	N18—C17—H17	117.2
C42—N43—Cu1	124.9 (3)	C17—N18—Cu2	125.4 (3)
C42—N43—C44	121.8 (4)	C17—N18—C19	121.2 (4)
C44—N43—Cu1	113.2 (2)	C19—N18—Cu2	113.0 (2)
N43—C44—H44	108.2	N18—C19—H19	106.3
N43—C44—C45	113.6 (3)	N18—C19—C20	117.4 (3)
N43—C44—C49	108.7 (3)	N18—C19—C24	107.8 (3)
C45—C44—H44	108.2	C20—C19—H19	106.3
C49—C44—H44	108.2	C20—C19—C24	112.0 (4)
C49—C44—C45	109.9 (3)	C24—C19—H19	106.3
C44—C45—H45A	108.0	C19—C20—H20A	109.2
C44—C45—H45B	108.0	C19—C20—H20B	109.2
H45A—C45—H45B	107.3	C19—C20—C21	112.0 (3)
C46—C45—C44	117.1 (3)	H20A—C20—H20B	107.9
C46—C45—H45A	108.0	C21—C20—H20A	109.2
C46—C45—H45B	108.0	C21—C20—H20B	109.2
C45—C46—H46	108.2	C20—C21—H21	108.5
C45—C46—C48	109.3 (4)	C20—C21—C22	111.1 (4)
C47—C46—C45	112.1 (3)	C22—C21—H21	108.5
C47—C46—H46	108.2	C23—C21—C20	110.4 (4)
C47—C46—C48	110.6 (4)	C23—C21—H21	108.5
C48—C46—H46	108.2	C23—C21—C22	109.9 (4)
C46—C47—H47A	109.5	C21—C22—H22A	109.5
C46—C47—H47B	109.5	C21—C22—H22B	109.5
C46—C47—H47C	109.5	C21—C22—H22C	109.5
H47A—C47—H47B	109.5	H22A—C22—H22B	109.5
H47A—C47—H47C	109.5	H22A—C22—H22C	109.5
H47B—C47—H47C	109.5	H22B—C22—H22C	109.5
C46—C48—H48A	109.5	C21—C23—H23A	109.5
C46—C48—H48B	109.5	C21—C23—H23B	109.5
C46—C48—H48C	109.5	C21—C23—H23C	109.5
H48A—C48—H48B	109.5	H23A—C23—H23B	109.5
H48A—C48—H48C	109.5	H23A—C23—H23C	109.5
H48B—C48—H48C	109.5	H23B—C23—H23C	109.5
O50—C49—C44	117.9 (4)	O25—C24—C19	118.5 (4)
O50—C49—O51	123.8 (4)	O25—C24—O26	124.0 (4)
O51—C49—C44	118.3 (4)	O26—C24—C19	117.5 (4)
C49—O51—Cu1	115.1 (3)	C24—O26—Cu2	115.2 (3)
C31—O52—Cu1	127.7 (3)	C6—O27—Cu2	127.1 (3)
N59—C58—H58	126.6	C54—N53—Cu2	128.3 (3)
C62—C58—H58	126.6	C57—N53—Cu2	125.9 (3)
C62—C58—N59	106.8 (4)	C57—N53—C54	105.4 (3)
C58—N59—H59	130 (4)	N53—C54—H54	125.3
C60—N59—C58	107.4 (4)	C55—C54—N53	109.4 (4)
C60—N59—H59	122 (4)	C55—C54—H54	125.3
N59—C60—H60	124.3	C54—C55—H55	126.9
N61—C60—N59	111.3 (4)	C54—C55—N56	106.2 (4)
N61—C60—H60	124.3	N56—C55—H55	126.9
C60—N61—Cu1	125.7 (3)	C55—N56—H56	119 (4)
C60—N61—C62	106.2 (3)	C57—N56—C55	108.0 (4)
C62—N61—Cu1	128.2 (3)	C57—N56—H56	133 (4)
C58—C62—N61	108.3 (4)	N53—C57—H57	124.5
C58—C62—H62	125.8	N56—C57—N53	111.1 (4)
N61—C62—H62	125.8	N56—C57—H57	124.5
			
Cu1—N43—C44—C45	−120.7 (3)	C62—C58—N59—C60	−0.2 (5)
Cu1—N43—C44—C49	1.9 (4)	Cu2—N18—C19—C20	−142.7 (3)
Cu1—N61—C62—C58	−178.2 (3)	Cu2—N18—C19—C24	−15.1 (4)
C28—C29—C30—C31	0.3 (7)	Cu2—N53—C54—C55	173.8 (3)
C28—N34—N35—C36	178.3 (3)	Cu2—N53—C57—N56	−174.2 (3)
C29—C28—C33—C32	−1.3 (6)	C3—C4—C5—C6	−0.7 (6)
C29—C28—N34—N35	−2.7 (6)	C3—C4—C5—C17	175.0 (4)
C29—C30—C31—C32	−2.1 (6)	C3—N9—N10—C11	−178.8 (3)
C29—C30—C31—O52	177.9 (4)	C4—C3—C8—C7	−1.3 (6)
C30—C31—C32—C33	2.1 (6)	C4—C3—N9—N10	179.3 (4)
C30—C31—C32—C42	−179.0 (4)	C4—C5—C6—C7	−0.5 (6)
C30—C31—O52—Cu1	−179.3 (3)	C4—C5—C6—O27	179.3 (4)
C31—C32—C33—C28	−0.5 (6)	C4—C5—C17—N18	−179.8 (4)
C31—C32—C42—N43	−3.1 (7)	C5—C6—C7—C8	0.8 (6)
C32—C31—O52—Cu1	0.6 (6)	C5—C6—O27—Cu2	−0.1 (6)
C32—C42—N43—Cu1	3.1 (6)	C5—C17—N18—Cu2	1.1 (6)
C32—C42—N43—C44	178.9 (4)	C5—C17—N18—C19	172.9 (4)
C33—C28—C29—C30	1.4 (6)	C6—C5—C17—N18	−4.4 (6)
C33—C28—N34—N35	178.4 (4)	C6—C7—C8—C3	0.1 (7)
C33—C32—C42—N43	175.8 (4)	C7—C6—O27—Cu2	179.6 (3)
N34—C28—C29—C30	−177.5 (4)	C8—C3—C4—C5	1.6 (6)
N34—C28—C33—C32	177.7 (4)	C8—C3—N9—N10	−0.6 (6)
N34—N35—C36—C37	−13.1 (6)	N9—C3—C4—C5	−178.3 (4)
N34—N35—C36—C41	167.9 (4)	N9—C3—C8—C7	178.6 (4)
N35—C36—C37—C38	178.9 (4)	N9—N10—C11—C12	−174.9 (4)
N35—C36—C41—C40	−179.5 (4)	N9—N10—C11—C16	4.5 (6)
C36—C37—C38—C39	1.3 (8)	N10—C11—C12—C13	178.2 (4)
C37—C36—C41—C40	1.5 (7)	N10—C11—C16—C15	−177.9 (4)
C37—C38—C39—C40	0.4 (8)	C11—C12—C13—C14	−0.1 (6)
C38—C39—C40—C41	−1.1 (7)	C12—C11—C16—C15	1.4 (6)
C39—C40—C41—C36	0.2 (7)	C12—C13—C14—C15	1.2 (7)
C41—C36—C37—C38	−2.2 (7)	C13—C14—C15—C16	−0.9 (7)
C42—C32—C33—C28	−179.4 (4)	C14—C15—C16—C11	−0.4 (7)
C42—N43—C44—C45	63.1 (5)	C16—C11—C12—C13	−1.2 (6)
C42—N43—C44—C49	−174.3 (3)	C17—C5—C6—C7	−175.9 (4)
N43—Cu1—O52—C31	−0.5 (4)	C17—C5—C6—O27	3.9 (6)
N43—C44—C45—C46	−94.6 (4)	C17—N18—C19—C20	44.5 (5)
N43—C44—C49—O50	176.3 (3)	C17—N18—C19—C24	172.1 (4)
N43—C44—C49—O51	−4.5 (5)	N18—C19—C20—C21	−147.5 (4)
C44—C45—C46—C47	−60.6 (5)	N18—C19—C24—O25	−169.4 (4)
C44—C45—C46—C48	176.4 (4)	N18—C19—C24—O26	12.6 (5)
C44—C49—O51—Cu1	5.0 (4)	C19—C20—C21—C22	83.6 (5)
C45—C44—C49—O50	−58.9 (5)	C19—C20—C21—C23	−154.3 (4)
C45—C44—C49—O51	120.3 (4)	C19—C24—O26—Cu2	−4.1 (5)
C49—C44—C45—C46	143.4 (4)	C20—C19—C24—O25	−38.7 (5)
O50—C49—O51—Cu1	−175.9 (3)	C20—C19—C24—O26	143.3 (4)
O52—C31—C32—C33	−177.9 (4)	C24—C19—C20—C21	86.9 (4)
O52—C31—C32—C42	1.0 (7)	O25—C24—O26—Cu2	178.0 (3)
C58—N59—C60—N61	0.4 (5)	O27—C6—C7—C8	−179.0 (4)
N59—C58—C62—N61	−0.1 (5)	N53—C54—C55—N56	0.0 (5)
N59—C60—N61—Cu1	178.1 (3)	C54—N53—C57—N56	−0.5 (5)
N59—C60—N61—C62	−0.5 (5)	C54—C55—N56—C57	−0.3 (5)
C60—N61—C62—C58	0.3 (5)	C55—N56—C57—N53	0.5 (5)
N61—Cu1—O52—C31	−179.5 (4)	C57—N53—C54—C55	0.3 (5)

### Hydrogen-bond geometry (Å, º)

**Table:**

*D*—H···*A*	*D*—H	H···*A*	*D*···*A*	*D*—H···*A*
N56—H56···O50	0.83 (5)	1.92 (6)	2.730 (5)	169 (5)
N59—H59···O25	0.90 (4)	1.83 (4)	2.726 (5)	175 (3)
C55—H55···O25^i^	0.95	2.38	3.316 (5)	168
C58—H58···O50^ii^	0.95	2.35	3.208 (6)	150

Symmetry codes: (i) −*x*+1, *y*−1/2, −*z*+1; (ii) −*x*, *y*+1/2, −*z*+1.
