# Supplementary figures and images for: Crystal structure and Hirshfeld surface analysis of (1H-imidazole-κN 3)[4-methyl-2-({[2-oxido-5-(2-phenyl­diazen-1-yl)phen­yl]methyl­idene}amino)penta­noate-κ3 O,N,O′]copper(II)
Source: Acta Crystallogr E Crystallogr Commun. 2024 Apr 11;80(Pt 5):468–71. doi: 10.1107/S2056989024002986 (PMC11074583; doi:10.1107/S2056989024002986)

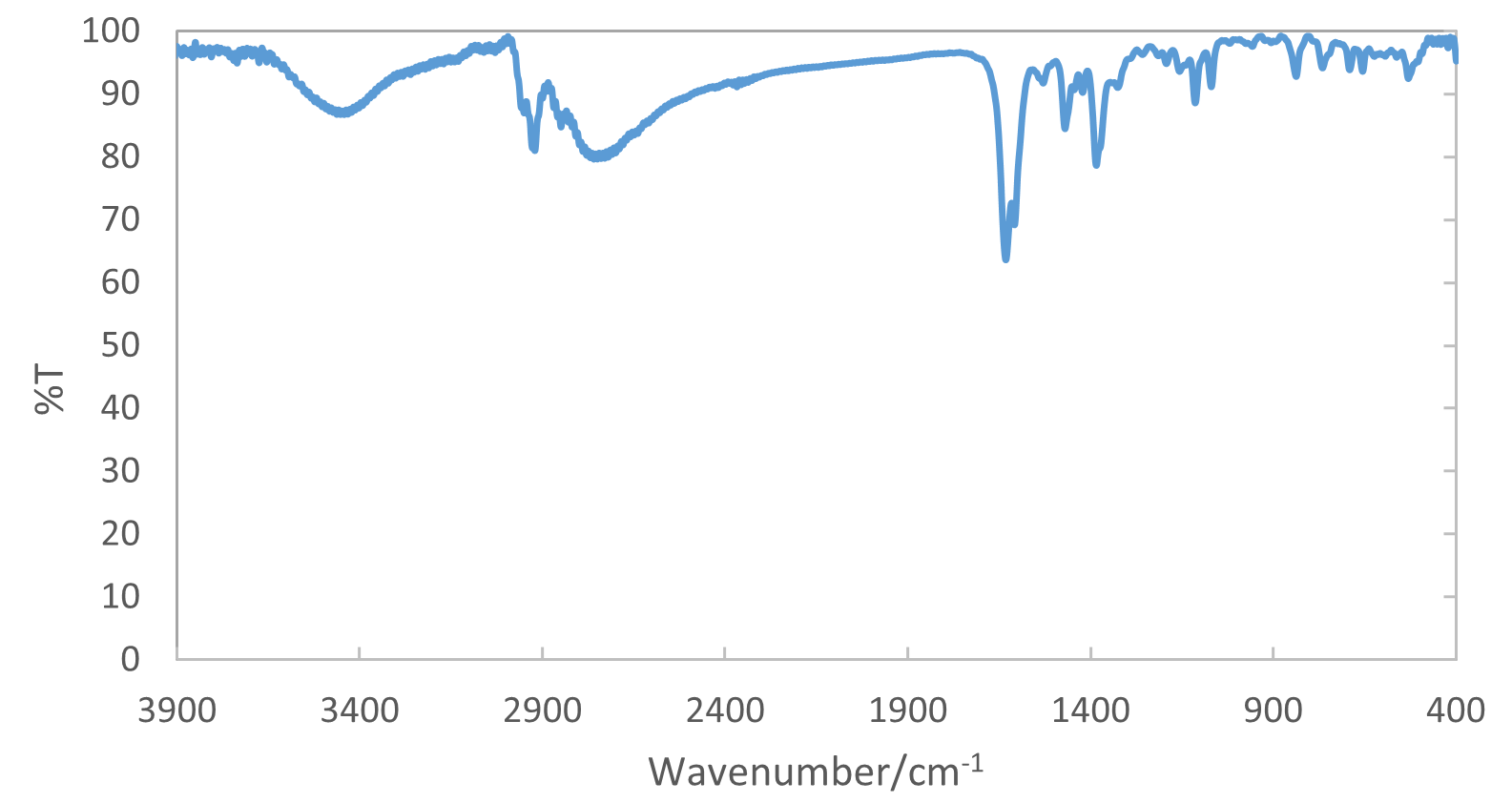

Supplement: Supplementary file 3 [file e-80-00468-sup3.tif]

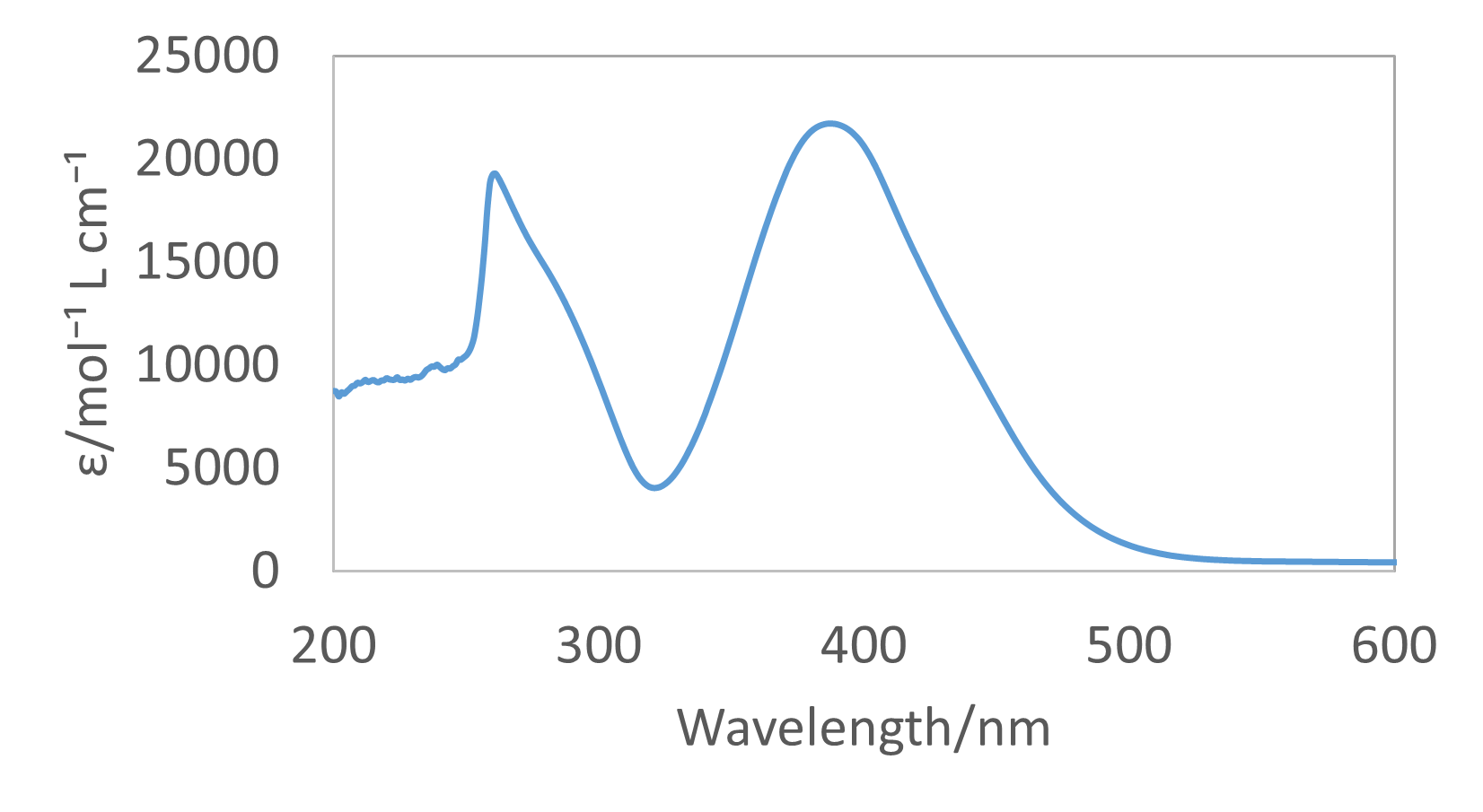

Supplement: Supplementary file 4 [file e-80-00468-sup4.tif]

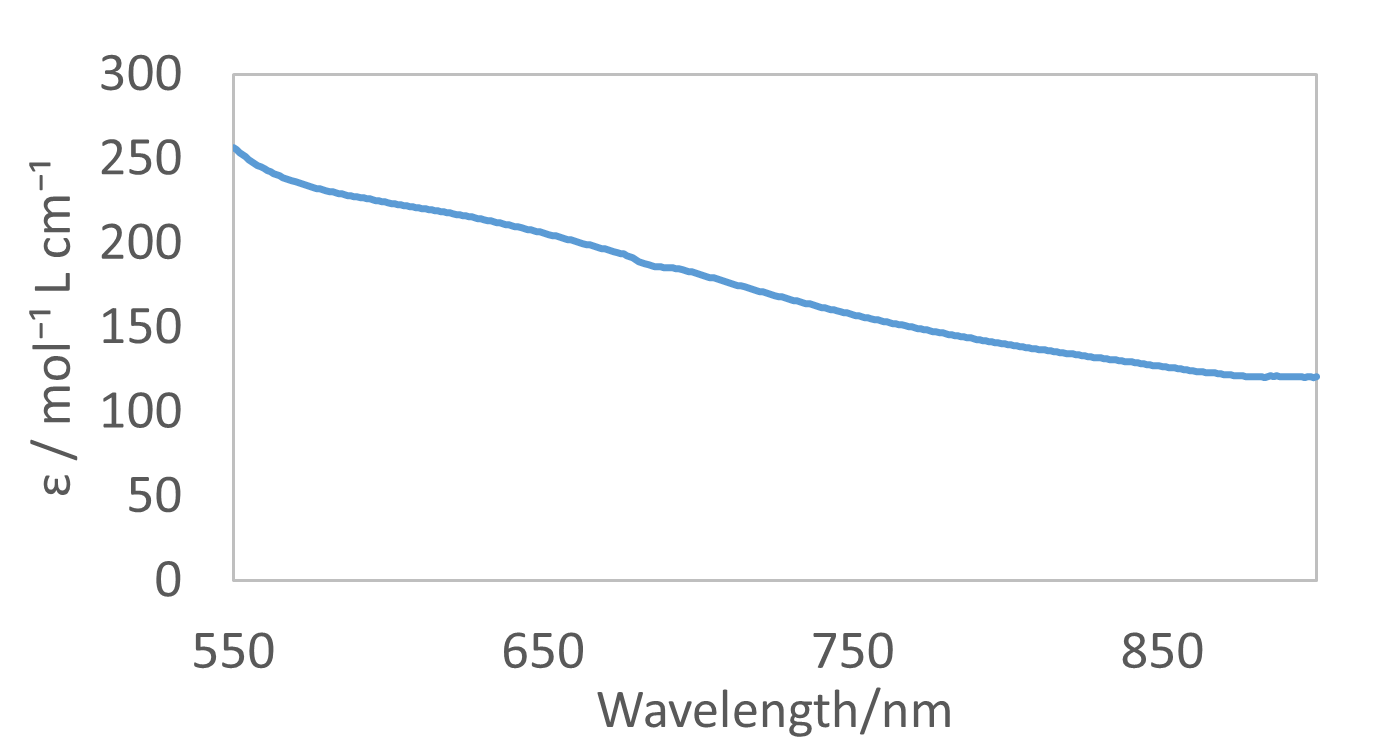

Supplement: Supplementary file 5 [file e-80-00468-sup5.tif]

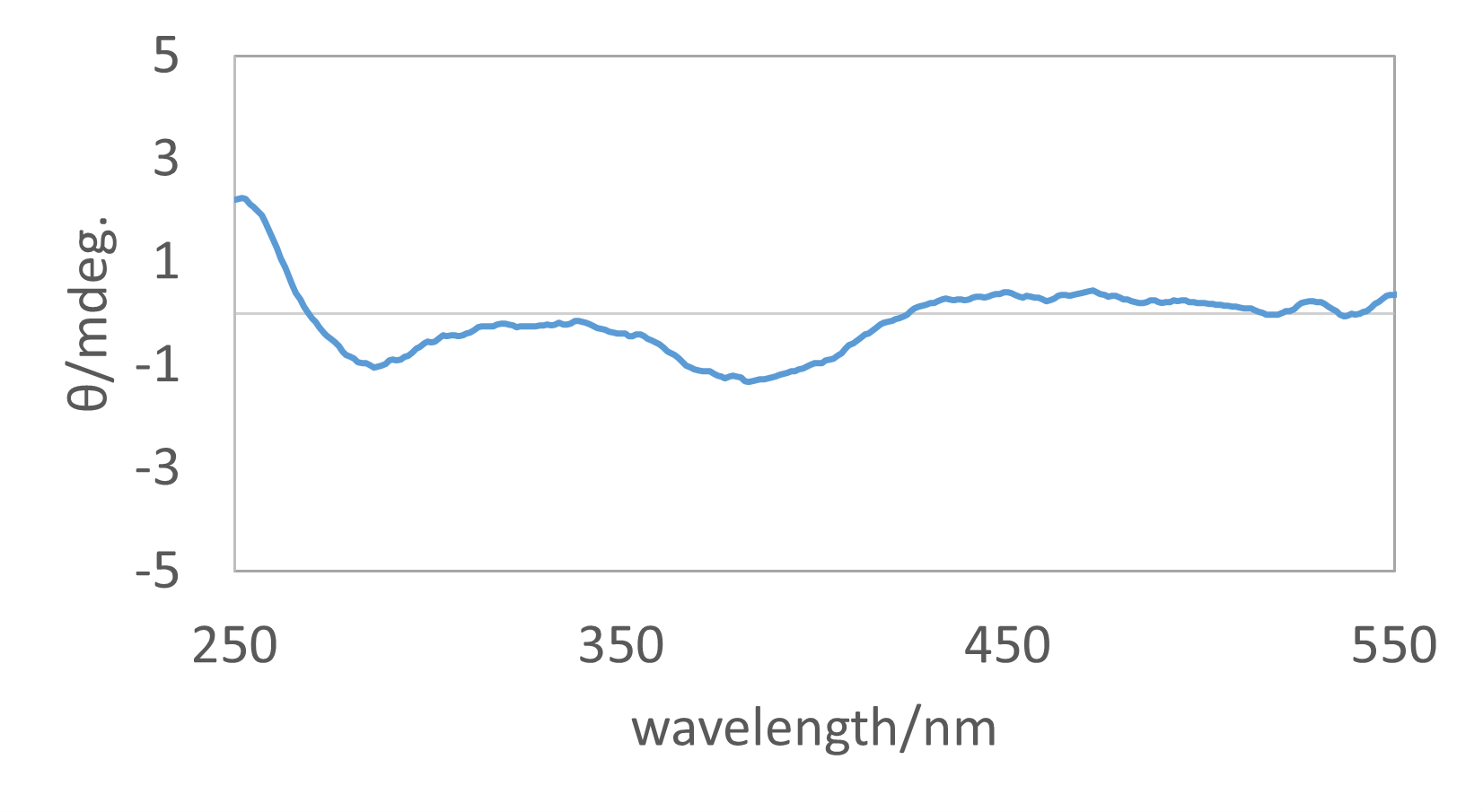

Supplement: Supplementary file 6 [file e-80-00468-sup6.tif]
